# The potential of key Alzheimer’s plasma biomarkers to mimic tau PET MUBADA-based disease staging

**DOI:** 10.1186/s13195-026-02085-6

**Published:** 2026-06-30

**Authors:** Inge M. W. Verberk, Lotte A. de Koning, Emma M. Coomans, Calvin Trieu, Anna E. Leeuwis, Jacob Hunter, Lee Honigberg, Wiesje M. van der Flier, Everard G. B. Vijverberg, Rik Ossenkoppele, Elsmarieke van de Giessen, Charlotte E. Teunissen

**Affiliations:** 1 Department of Laboratory Medicine, Neurochemistry Laboratory, Amsterdam University Medical Centers, Vrije Universiteit Amsterdam, De Boelelaan 1118, Amsterdam, 1081 HZ The Netherlands; 2 Department of Radiology and Nuclear medicine, Amsterdam UMC, Vrije Universiteit Amsterdam, Amsterdam, The Netherlands; 3 Department of Neurology, Alzheimer Center, Amsterdam UMC, Vrije Universiteit Amsterdam, Amsterdam, The Netherlands; 4 Amsterdam Neuroscience, Neurodegeneration, Amsterdam, The Netherlands; 5 Department of Medical Psychology, Amsterdam UMC, Vrije Universiteit Amsterdam, Amsterdam, The Netherlands; 6 Amsterdam Neuroscience, Brain Imaging, Amsterdam, The Netherlands; 7 ALZpath Inc, California, USA; 8 Alzheimer Nederland, Amersfoort, The Netherlands; 9 Epidemiology and Data Science, Vrije Universiteit Amsterdam, Amsterdam UMC location VUmc, Amsterdam, The Netherlands; 10 Amsterdam Public Health, Amsterdam, The Netherlands

**Keywords:** Alzheimer’s disease, Tau staging, Tau positron emission tomography, Plasma biomarkers, Cognition

## Abstract

**Background:**

Patients with Alzheimer’s disease (AD) with a low to intermediate tau-PET burden might benefit most from anti-amyloid treatment. Staging tau burden with plasma biomarkers would offer a scalable alternative to staging with tau-PET. This study investigated whether key plasma biomarkers P-tau217, P-tau181, Aβ42/40, GFAP and NfL can be used to accurately stage amyloid status (A-/A+) and tau-PET burden, and evaluated the relation of such a plasma-based staging system with cognitive outcomes over time.

**Methods:**

We included 105 participants with subjective cognitive decline (*n* = 27 A-, *n* = 18 A+), A+ mild cognitive impairment (*n* = 10) or A + AD-dementia (*n* = 50) from the Amsterdam Dementia Cohort who underwent [^18^F]flortaucipir PET-burden assessment (T_low_, T_intermediate_ or T_high_; based on MUBADA SUVr) and longitudinal cognitive assessment (average follow-up: 4.1 ± 3.5 years). AD-related plasma biomarkers were measured with Simoa. Discriminative performance (AUC) of each marker was compared using ROC analysis, and combined utility was assessed with logistic regression. Subsequently, cutoffs were established aiming for 90%-specificity, to regroup participants into a plasma-based staging scheme. Age-, sex- and education-adjusted linear mixed models (LMM) were performed to compare associations of plasma versus PET-based staging with longitudinal cognition.

**Results:**

27 participants were A-T_PET_low_, 22 A+T_PET_low_, 27 A+T_PET_int_ and 29 A+T_PET_high_. To discriminate A-T_PET_low_ participants from A+T_PET_low/int_ participants, P-tau217 performed best among all measured markers P-tau217, P-tau181, Aβ42/40, GFAP and NfL (AUC = 0.92 [95% CI: 0.856–0.985]). To discriminate A+T_PET_high_ participants from A+T_PET_low/int_ participants, also P-tau217 performed best among all markers (AUC = 0.74 [95% CI: 0.618–0.862]). A combination of Wald’s backward-selected plasma markers did not statistically improve discriminative performance (DeLong’s *p* > 0.05; two-marker combinations selected). Applying two cutoffs for P-tau217 as well as for the two-marker combinations, at 90% specificity to discriminate subsequent groups, we derived two plasma-based staging schemes. While the tau-PET staging scheme significantly and consistently associated with cognitive performance and decline across cognitive domains in LMMs, the plasma staging schemes did not.

**Conclusions:**

Performance of plasma-based staging approaches developed in this study were high when discriminating individuals without amyloid pathology, while this was moderate when discriminating amyloid positive individuals with a high tau-PET burden. Our LMM findings visualize that tau staging in amyloid-positive individuals remains optimally performed with tau-PET scans.

**Supplementary Information:**

The online version contains supplementary material available at https://doi.org/10.1186/s13195-026-02085-6.

## Background

Alzheimer’s disease (AD) pathophysiology is characterized by the cerebral accumulation of amyloid-β (Aβ)-containing plaques and hyperphosphorylated tau (P-tau)-containing tangles, which eventually results in cognitive decline to dementia [1, 2]. First Aβ-targeting therapies have shown to alter the AD pathophysiology as well as the clinical course [3–5] and are now reaching clinical care. These therapies are likely most effective when initiated in early disease stages [3–5]. Particularly Donanemab has been shown most effective in individuals with evidence of cerebral Aβ accumulation who still have a low to intermediate cerebral tau burden [5]. The Aβ-targeting therapies are costly and come with a risk of adverse events. Therefore, accurately identifying individuals who are expected to have the most favorable treatment benefit-to-risk ratio would aid clinical decision making.

To identify individuals with AD who likely respond favorably to anti-amyloid treatment, positron emission tomography (PET) scans visualizing cerebral Aβ accumulation and tau burden stage can be used [1, 6–8]. In addition, proof-of-concept for accurate staging using cerebrospinal fluid (CSF) markers has recently been achieved [9]. More affordable and scalable methods to assess treatment eligibility are needed to enable widescale adoption of the therapeutics in dementia care. A blood test incorporating the Alzheimer’s-related plasma biomarkers P-tau, Aβ42/40, glial fibrillary acidic protein (GFAP) and/or neurofilament light (NfL) might be suitable to simultaneously determine amyloid pathology and tau burden stage [10–12]. Previous studies indicate that these biomarkers indeed change along with tau-PET burden stages, with different magnitudes of change and diagnostic accuracies for tau burden for each of the markers [8, 13–17]. These previous studies have used various tau-PET burden staging approaches (based on Braak topographic distributions [8, 14, 15, 17] or based on semi-quantitative values in other composite brain regions [13, 15]). Limited studies have investigated plasma biomarker staging using the Donanemab tau-PET staging method (*i.e.*, in the multiblock barycentric discriminant analysis (MUBADA) region) [13]. Also, limited studies have assessed the association between plasma-based staging classifications and clinical course [8, 18]. In addition, different combinations of plasma biomarkers, both innovative and more established, and measured with various technologies including less-scalable technologies such as targeted mass-spectrometry, were assessed [8, 13–15, 17, 18]. Independent repetition of the previous studies’ findings utilizing a scalable lab technology, accompanied with an investigation of relationships of a plasma-based Aβ status and tau burden staging with relevant clinical outcomes, such as longitudinal cognitive decline, is warranted.

Here, we aimed to examine if (a combination of) the plasma biomarkers P-tau217, P-tau181, Aβ42/40 ratio, GFAP and NfL can be used to stage Aβ status and tau-PET burden. In addition, we validated the relation of a plasma-based versus a PET-based AD staging approach with clinical course, by comparing the associations of the plasma-based versus PET-based staging approaches with subsequent longitudinal cognitive trajectories.

## Methods

### Cohort

From the Amsterdam Dementia Cohort [19, 20] and SCIENCe project [21] we selected 105 individuals with a baseline diagnosis of subjective cognitive decline (SCD) (*n* = 45; 27 (60%) normal amyloid status, 18 (40%) abnormal amyloid status), mild cognitive impairment (MCI) due to AD (*n* = 10; all abnormal amyloid status) or probable AD (*n* = 50; all abnormal amyloid status) with (1) amyloid status determined with PET or CSF, (2) available [^18^F]-flortaucipir tau-PET scans, and (3) a plasma sample in our biobank collected within 13 months of the tau-PET scan (median 5.0 months; interquartile range 4.3 months). The study cohort selection and study procedures are described in more detail elsewhere [22, 23]. In short, all participants of the current study underwent a standardized dementia screening including physical, neurological, and neuropsychological examination, standard blood assessment, APOEε4 genotyping, CSF AD biomarker analysis and/or an amyloid PET scan electroencephalography, and brain MRI [19, 20]. MCI and AD-dementia diagnoses were established in a multidisciplinary consensus meeting according to the NIA-AA criteria [24, 25] and SCD was assigned when normal results were obtained on all clinical and cognitive tests, and when there was no evidence of other neurological or psychiatric diseases causing cognitive worries [26].

### PET or CSF-based amyloid status

Amyloid status (dichotomous: normal (-) or abnormal (+)) was determined using amyloid-PET scans (*n* = 64; *n* = 19 with [^18^F]Florbetaben, *n* = 43 with [^18^F]Florbetapir, *n* = 2 with [^11^C]PiB) or with cerebrospinal fluid (CSF) AD biomarker analysis (*n* = 41; *n* = 20 with Innotest ELISA, Fujirebio, Belgium, *n* = 17 with Elecsys, Roche Diagnostics GmbH, Germany, *n* = 4 with results obtained elsewhere and taken from the patient file). Amyloid PET scans were performed according to procedures described elsewhere [21, 27–31]. Scans were visually rated as normal or abnormal by a nuclear medicine physician according to company guidelines, or in case of [^11^C]PiB scan according to previously published methods [31]. The measured CSF AD biomarkers included Aβ42, P-tau181 and t-tau. Innotest Fujirebio P-tau181/Aβ42 ratio > 0.06 [23] (note: Aβ42 values were first drift-corrected [32]) and Elecsys Roche P-tau181/Aβ42 ratio > 0.02 were considered to reflect an abnormal (+) amyloid status [33].

### Tau-PET scanning and staging

Dynamic [^18^F]Flortaucipir PET scans were acquired on a Philips Ingenuity TF-64 PET/CT scanner (Philips Medical Systems, the Netherlands) after bolus net injection of approximately 240 MBq [34, 35]. Low-dose CT scans were used for attenuation correction of the PET scan. Using 3D RAMLA with a matrix size of 128 × 128 × 90 and a final voxel size of 2 × 2 × 2 mm [3], PET data were reconstructed. Standard corrections for dead time, decay, attenuation, random and scatter were performed. Participants were also scanned on a 3.0T MRI scanner to obtain a three-dimensional T1 weighted sequence which was co-registered to the tau-PET scan to extract regional tau-PET uptake values.

We extracted standardized uptake value ratio (SUVr) from summed tau-PET frames (80–100 min post-injection) in a global weighted MUBADA volume of interest (VOI) according to published methods [36–38] and using subject-specific white matter reference regions derived by the parametric estimation of reference signal intensity (PERSI) method [36, 37]. The MUBADA VOI was used in recent TRAILBLAZER-ALZ and TRAILBLAZER-ALZ2 clinical trials for classification and inclusion of participants, and comprises voxels from all brain lobes, however more weight is given to voxels in the posterior and lateral temporal lobe, parietal lobe and association regions of the occipital cortex. This approach is in agreement with the recent Alzheimer’s Association Revised criteria for Diagnosis and Staging, which recommends incorporating both topography and uptake magnitude in tau load staging [39]. In addition, it assigns higher weights in the left hemisphere [38]. Participants were stratified based on MUBADA SUVr into low (< 1.10), intermediate (1.10–1.46), or high (> 1.46) tau binding groups, and by visual examination of the tau-PET scan [3]. Visual examinations were performed according to the US food and drug administration-approved method [40] and as previously described [41], and were performed to check whether tau binding showed an AD-specific pattern.

### Blood biomarker analyses

Non-fasted EDTA plasma samples were obtained by venipuncture, centrifuged for 10 min at 1,800xg at room temperature, aliquoted into 0.5mL portions in polypropylene tubes, and stored at -80°C in the Amsterdam Dementia Biobank. Only one plasma sample per participant was included in this study. At time of analysis, samples were shortly thawed at room temperature in front of a cold-air fan and centrifuged for 10 min at 10,000xg. All sample measurements were performed on the Simoa HD-X analyzer. We used the Simoa neurology 4-plex E kit (pre-commercial pilot kit lot version, Quanterix, USA; first freeze-thaw cycle; as described elsewhere [23]), the Simoa P-tau181 V2 kit (kit lot # 103714, Quanterix, USA; second freeze-thaw cycle; as described elsewhere [22, 23]) and the prototype Simoa P-tau217 ALZpath assay (kit lot #MAB231122, ALZpath, USA; third freeze-thaw cycle). Assays were run according to manufacturer’s instructions. The neurology 4-plex E kit was run in singlicates, both P-tau assays were run in duplicates. Average intra-assay coefficients of variation (CV) of duplicate P-tau181 and P-tau217 measurements were < 10%, and between-run CVs of quality control samples were < 20% for all markers with all assays and all quality control samples. P-tau217 results were missing for 6 participants due to instrument errors (*n* = 3) and insufficient sample volume (*n* = 3), and Aβ42/40 result was missing for 1 participant due to severe hemolysis of the sample, which negatively impacts Aβ42 and Aβ40 results [42].

### Cognitive evaluation

Cognitive evaluation was performed at each annual clinical visit using the MMSE and an extensive battery of neuropsychological tests [19–21]. All test scores were standardized using baseline as a reference. Four main cognitive domains were constructed: memory, language, executive functioning and attention. The memory domain score consisted of the Dutch version of the Rey Auditory Verbal Learning Test (immediate and delayed recall) and the Visual Association Test A. The language domain score consisted of the category fluency test animals and Visual Association Test naming. The executive function domain score consisted of the Trail Making Test index (time spend on Trail Making Test B divided by the time spend on Trail Making Test A), and Stroop index (time spend on card III (color-word naming) divided by the averaged time spend on card I (word naming) and II (color naming)). The attention domain score consisted of the Trail Making Test A, Stroop card I, and Stroop card II tests. Trail Making Test and Stroop scores were inverted so that lower scores equaled poorer test results.

For each domain, the standardized test scores at each visit were averaged, when at least two contributing test scores were available, or otherwise it was set to missing. Subsequently, a global cognition score was constructed by averaging the four cognitive domain scores, which was set to missing when more than one domain score was missing.

We included neuropsychological test scores from both before and after tau-PET imaging to accurately map the participants’ cognitive slopes. Test score closest to the tau-PET date was considered the baseline (range time interval between baseline test score and tau-PET date: -317 to + 197 days). We have a total number of 465 cognitive evaluations covering 4.1 ± 3.5 years (191 before the tau-PET scan; 274 after the tau-PET scan) of our 105 participants (range 1–11 evaluations per participant; median: 4 visits; *n* = 92 > 1 visit). Across the evaluations, memory domain score was missing in 36 (7.7%), language domain score was missing in 83 (17.8%), executive functioning domain score was missing in 90 (19.4%), attention domain score was missing in 85 (18.3%), and global cognition score was missing in 105 (22.6%) out of 465.

### Statistical analysis

We performed statistical data analysis using RStudio version 4.4.1 (packages: *pROC*, *lme4*) and considered *p* < 0.05 as statistically significant. Plasma levels were natural log (Ln)-transformed prior to all analyses to meet data normality assumptions.

Cohort demographics and clinical characteristics were compared between A(myloid)T(au)_PET_stages_ groups with chi-squared tests, analysis of variance or Kruskal-Wallis tests as appropriate. Plasma biomarker differences between AT_PET_stages_ groups were assessed with age- and sex-adjusted analyses of covariance. Bonferroni correction was applied to correct for multiple testing. Receiver operating characteristics (ROC) curves among individual plasma markers were constructed to obtain area under the curves (AUCs). In addition, backward logistic regression based on Wald’s *p*-statistics was conducted amongst the plasma biomarkers to select optimal marker combinations to discriminate AT_PET_stages_ groups (groups resulting from tau-PET MUBADA SUVr stratification). Collinearity across the plasma biomarker results was excluded, by assessing the variance inflation factor, which was below the acceptable cutoff of 5. Spearman’s correlations coefficients were all below the acceptable cutoff of 0.7, except between P-tau217 and P-tau181 (Spearman’s rho = 0.83). ROC curves of marker combinations were compared to ROC curves of individuals markers using the DeLong’s test. Plasma-based staging was constructed using the best performing plasma biomarker and plasma biomarker combinations, at the thresholds corresponding to 90% specificity levels aiming to effectively exclude those that are less likely to benefit from treatment. This 90% specificity threshold was chosen according to the recommendations from the Global CEO Initiative [43]. For plasma biomarker combinations, predicted values based on logistic regression fits were used. A Sankey plot was created to compare the original AT_PET_stages_ with the newly established AT_plasma_stages_ groupings. In addition, linear mixed models were performed to compare the clinical relevance of the AT_PET_stages_ versus the AT_plasma_stages_ groupings in association with concurrent and longitudinal cognition scores. Models included subject-specific intercepts. Random slopes were added if it improved the model fit based on the Akaike information criterion using χ^2^ statistics. Models included terms for group (AT_PET_stages_ or AT_plasma_stages_), age, sex, education, time (tau-PET date as t = 0), and an interaction term of group *x* time as fixed variables, and cognition scores as dependent variable (separate models per score). *P*-values of the linear mixed models were adjusted for multiple comparisons using the 10% false discovery rate (FDR) procedure [44].

## Results

### Participants

We included 105 participants, of whom 27 were Aβ negative and tau-PET burden low (A-T_PET_low_; all SCD), 22 were Aβ + and tau-PET burden low (A+T_PET_low_; 13 SCD, 2 MCI, 7 dementia), 27 were Aβ + and tau-PET burden intermediate (A+T_PET_int_; 4 SCD, 6 MCI, 17 dementia), and 29 were Aβ + and tau-PET burden high (A+T_PET_high_; 1 SCD, 2 MCI, 26 dementia). A summary of the cohort’s demographics and clinical characteristics is presented in Table 1. A+T_PET_high_ participants were significantly younger than A+T_PET_low_ and A+T_PET_int_ participants (both: *p* < 0.005). Distribution of sex and education was similar across all groups. The A-T_PET_low_ group had fewer ApoEε4 carriers compared to the three A+ groups (all *p* < 0.05).

**Table 1:** Demographics and clinical characteristics

Characteristic	A-T_PET_low_ (*n* = 27)	A+T_PET_low_ (*n* = 22)	A+T_PET_int_ (*n* = 27)	A+T_PET_high_ (*n* = 29)	*p*-value
Diagnosis (SCD/MCI/dementia)	27/0/0^b, c, d^	13/2/7^a, c, d^	4/6/17^a, b^	1/2/26^a, b^	< 0.001
Age, y	65 (8)	69 (7)^d^	68 (6)^d^	62 (7)^b, c^	0.001
Female sex, *n* (%)	14 (52)	7 (32)	11 (41)	19 (66)	0.086
Education score	5.3 (1.3)	5.6 (0.8)	5.8 (1.2)	5.6 (1.0)	0.454
ApoEε4 carrier, *n* (%)	6 (22)^b, c, d^	15 (71)^a^	21 (81)^a^	18 (62)^a^	< 0.001
MMSE score	29 (1)	27 (3)	25 (4)	22 (4)	< 0.001
[^18^F]flortaucipir (tau)-PET					
MUBADA SUVr	1.02 (0.03)	1.05 (0.04)	1.25 (0.14)	1.68 (0.19)	< 0.001
Plasma biomarkers					
P-tau217, pg/mL	0.33 (0.15)^b, c, d^	0.91 (0.46)^a, d^	1.15 (0.51)^a^	1.55 (0.63)^a, b^	< 0.001
P-tau181, pg/mL	1.57 (0.61)^b, c, d^	2.72 (1.40)^a^	2.79 (0.88)^a^	3.06 (0.97)^a^	< 0.001
Aβ42/40 ratio	0.06 (0.01)^b, c, d^	0.05 (0.01)^a^	0.05 (0.01)^a^	0.05 (0.01)^a^	0.003
GFAP, pg/mL	93.5 (40)^b, c, d^	148 (81)^a^	159 (65)^a^	158 (52)^a^	< 0.001
NfL, pg/mL	14.1 (5.6)^d^	18.4 (6.6)	17.6 (5.0)	17.3 (4.5)^a^	0.026
Cognition, z-scores					
Global cognition	0.67 (0.3)^c, d^	0.32 (0.5)^c, d^	-0.22 (0.5)^a, b^	-0.33 (0.4)^a, b^	< 0.001
Memory domain	1.05 (0.4)^b, c, d^	0.10 (0.7)^a, d^	-0.41 (0.7)^a^	-0.60 (0.6)^a, b^	< 0.001
Language domain	0.61 (0.4)^c, d^	0.37 (0.5)^c, d^	-0.35 (0.8)^a, b^	-0.32 (0.8)^a, b^	< 0.001
Executive functioning domain	0.49 (0.5)^c, d^	0.20 (0.5)	-0.35 (0.8)^a^	-0.16 (0.7)^a^	< 0.001
Attention domain	0.51 (0.5)^d^	0.32 (0.6)^d^	0.05 (0.5)^d^	-0.60 (1.2)^a, b, c^	< 0.001

Data is shown as mean (SD) or *n* (%) for the amyloid status and tau-PET burden groups, at time of tau-PET scanning. Amyloid status (-/+) is according to cerebrospinal fluid testing or amyloid PET. Tau-PET burden (low, intermediate, high) is according to MUBADA SUVr with SUVr < 1.10 classified as low, SUVr 1.10–1.46 classified as intermediate and SUVr > 1.46 classified as high. Education score is according to the Verhage system (scale 1–7) [45]. Cognition is expressed as standardized domain z-scores, calculated by averaging standardized performance across the relevant tests within each domain. Global cognition was computed as the mean of the individual domain z-scores. Cognitive evaluation scores closest to tau-PET date are reported. Differences between groups were calculated using χ^2^ tests for categorical variables (diagnosis, sex, ApoEε4), and Kruskal Wallis tests (education), analysis of variance (age, MUBADA SUVr, cognitive tests), and analysis of variance covaried for age and sex (P-tau217, P-tau181, Aß42/40, GFAP, NfL) for continuous variables. ApoEε4 carriership was missing for two participants (one in A+T_PET_low_, one in A+T_PET_int_). Plasma P-tau217 level was missing for six participants (two in A+T_PET_low_, four in A+T_PET_high_). Plasma Aβ42/40 ratio was missing for one participant (in A+T_PET_low_)

*A* amyloid status, *T* tau, *PET* positron emission tomography, *int* intermediate, *SCD* subjective cognitive decline, *MCI* mild cognitive impairment, *ApoE* apolipoprotein E, *SUVr* standardized uptake value ratio, *P-tau* phosphorylated tau, *Aβ* amyloid-beta, *GFAP* glial fibrillary acidic protein, *NfL* neurofilament light

^a^ Significantly different from A-T_PET_low_

^b^ Significantly different from A+T_PET_low_

^c^ Significantly different from A+T_PET_int_

^d^ Significantly different from A+T_PET_high_

**Fig. 1:**
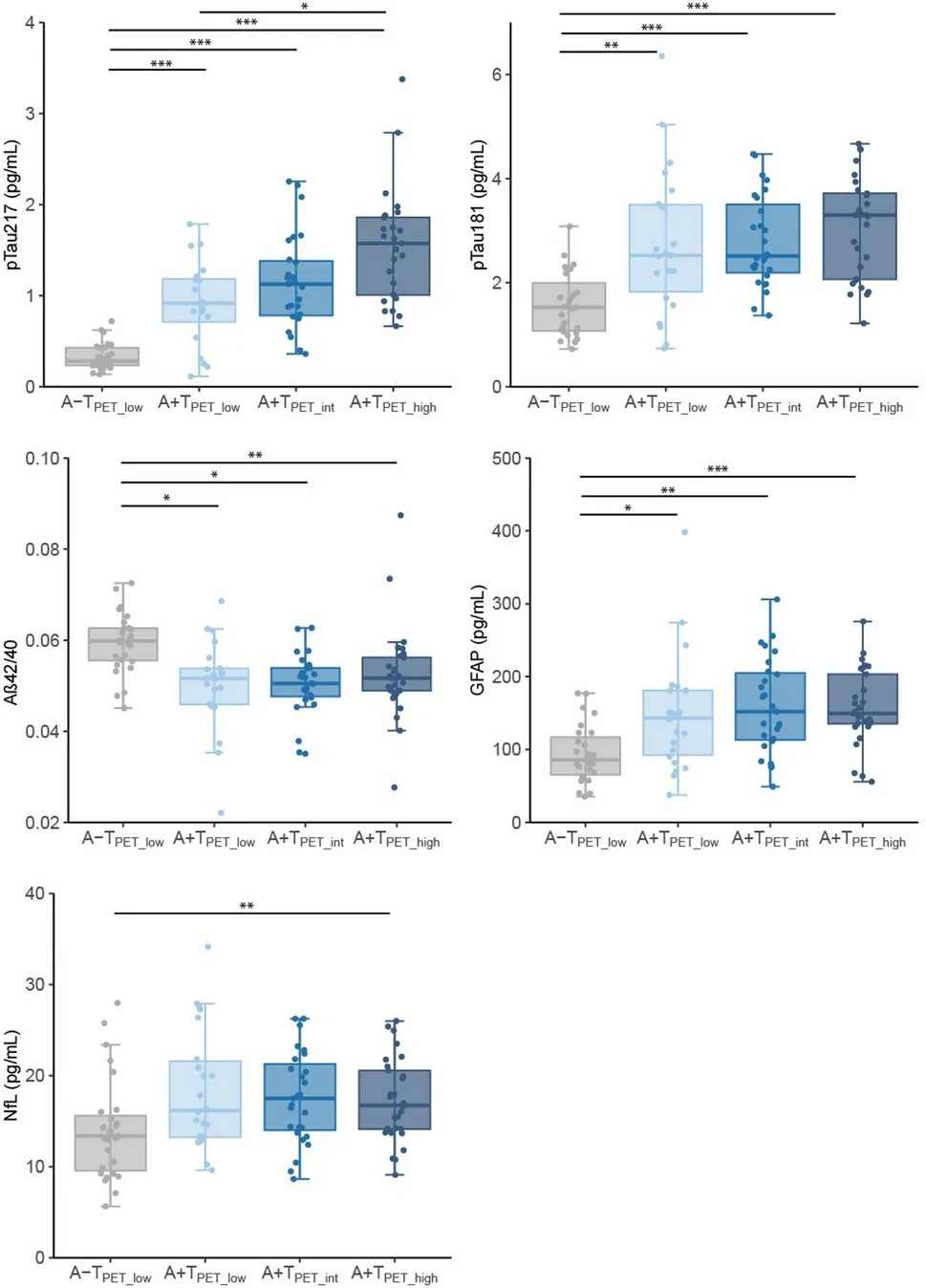
Boxplots of raw plasma biomarker values across the amyloid status and tau-PET groups. Amyloid status (-/+) is according to cerebrospinal fluid testing or amyloid PET. Tau-PET burden (low, intermediate, high) is according to MUBADA SUVr with SUVr < 1.10 classified as low, SUVr 1.10–1.46 classified as intermediate and SUVr > 1.46 classified as high. Differences between groups were calculated using analysis of variance covaried for age and sex, with Bonferroni correction for multiple comparisons. *A *amyloid status, *T* tau, *PET* positron emission tomography, *Int* intermediate, *P-tau* phosphorylated tau, *Aβ* amyloid beta, *GFAP* glial fibrillary acidic protein, *NfL *neurofilament light. *p < 0.05, **p < 0.01, ***p < 0.001

Plasma biomarker levels are visualized in Fig. 1. Compared to the A-T_PET_low_ group, plasma P-tau217, P-tau181 and GFAP were higher and plasma Aβ42/40 was lower in the A+ groups irrespective of Tau-PET burden stage (A+T_PET_low_: age/sex-corrected, all biomarkers *p* < 0.03; A+T_PET_int_: age/sex-corrected, all biomarkers *p* < 0.03; A+T_PET_high_: age/sex-corrected, all biomarkers *p* < 0.01). NfL was only significantly higher in the A+T_PET_high_ group compared to the A-T_PET_low_ group (age/sex-corrected: *p* = 0.001). Among the three A+ groups, only P-tau217 differed significantly, with higher values observed in the A+T_PET_high_ compared to the A+T_PET_low_ group (age/sex-corrected: *p* < 0.004). None of the plasma markers differed significantly between the A+T_PET_low_ and A+T_PET_int_ groups, or between the A+T_PET_int_ and A+T_PET_high_ groups. Given the similarity in plasma biomarker levels and in clinical benefit reported in the TRAILBLAZER-ALZ and TRAILBLAZER-ALZ2 trials across A+T_PET_low_ and A+T_PET_int_ participants, these two groups were pooled into a single A+T_PET_low/int_ group for subsequent analyses.

### Accuracy of the plasma biomarkers to discriminate A-T_PET_low_ and A+T_PET_high_ participants

**Fig. 2:**
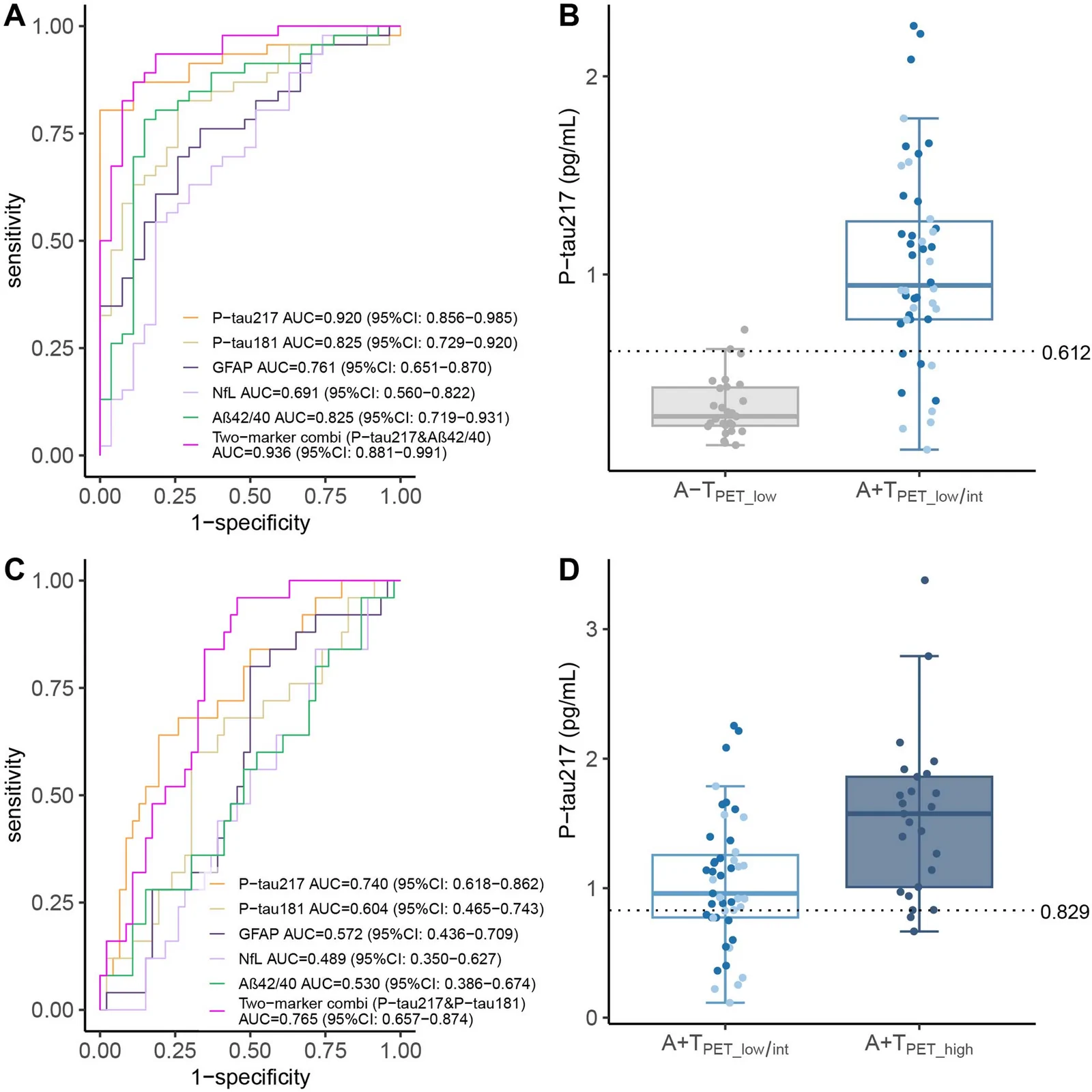
ROC curves and boxplots for A-T_PET_low_ vs. A+T_PET_low/int_ (top) and A+T_PET_high_ vs. A+T_PET_low/int_ (bottom). Receiver operating characteristic (ROC) area under the curves (AUCs) with 95% confidence intervals (CI) are presented for the plasma biomarkers and selected marker combinations based on Wald’s backward elimination logistic regression, to discriminate amyloid negative tau-PET burden low (A-T_PET_low_) from amyloid positive tau-PET burden low and intermediate (A+T_PET_low/int_) participants (top panel; two-marker combination included P-tau217 and Aß42/40), or to discriminate A+T_PET_low/int_ from amyloid positive tau-PET burden high (A+T_PET_high_) participants (bottom panel; two-marker combination included P-tau217 and P-tau181). Participants in the A+T_PET_low/int_ group are colored by their original T_PET_low_ (light blue) or T_PET_int_ (dark blue) status. Raw plasma P-tau217 values are plotted on the y-axis in graphs on the right and thresholds corresponding to 90% specificity levels are illustrated as dotted lines. ROC-AUCs were calculated only on participants with complete biomarker data. PET = positron emission tomography, P-tau = phosphorylated tau, Aβ = amyloid-beta, GFAP = glial fibrillary acidic protein, NfL = neurofilament light

We next assessed the potential of the plasma markers to discriminate A-T_PET_low_ participants from A+T_PET_low/int_ participants, and to discriminate A+T_PET_low/int_ participants from A+T_PET_high_ participants (Fig. 2).

AUCs of the individual plasma biomarkers to discriminate A-T_PET_low_ participants from A+T_PET_low/int_ participants ranged from 0.691 (NfL) to 0.920 (P-tau217). As comparison, age and sex had an AUC of 0.694 (95%CI: 0.553–0.835) to discriminate A-T_PET_low_ participants from A+T_PET_low/int_. A combination of biomarkers selected with Wald’s backward elimination regression did not meaningfully improve the discrimination of A-T_PET_low_ participants from A+T_PET_low/int_ participants, as compared to P-tau217 alone (selected combination: P-tau217 + Aβ42/40 ratio, AUC = 0.936 (95%CI: 0.881–0.991); ΔAUC = 0.015, DeLong’s *p* = 0.724). Of note, exploratively combining all 5 markers did not improve the discrimination of A-T_PET_low_ participants from A+T_PET_low/int_ participants compared to P-tau217 alone (AUC = 0.938, 95%CI: 0.881–0.995; ΔAUC = 0.018, DeLong’s *p* = 0.688).

AUCs of the individual plasma biomarkers to discriminate A+T_PET_high_ participants from A+T_PET_low/int_ participants ranged from 0.489 (NfL) to 0.740 (P-tau217). As comparison, age and sex had an AUC of 0.824 (95%CI: 0.714–0.934) to discriminate A+T_PET_high_ from A+T_PET_low/int_ participants. A combination of biomarkers selected with Wald’s backward elimination regression did not meaningfully improve the discrimination of A+T_PET_high_ participants from A+T_PET_low/int_ participants, as compared to P-tau217 alone (selected combination: P-tau217 + P-tau181, with AUC = 0.765 (95%CI: 0.657–0.874); ΔAUC = 0.025, DeLong’s *p* = 0.762). Of note, exploratively combining all 5 markers did not significantly improve the discrimination of A+T_PET_high_ participants from A+T_PET_low/int_ participants compared to P-tau217 alone (AUC = 0.797 (95%CI: 0.694–0.900); ΔAUC = 0.057; DeLong’s *p* = 0.482).

Participants were reclassified according to ≥ 90% specificity cutoffs. Given the limited statistical benefit of combining plasma marker results to discriminate tau-based PET stages, and to enhance clinical feasibility of a plasma-based staging, we first focused the reclassification of our participants on P-tau217. At a P-tau217 cutoff level > 0.612 pg/mL, specificity of ≥ 90% was achieved for excluding A-T_PET_low_, which resulted in a good sensitivity of 80% for identifying A+T_PET_low/int_ (Fig. 2B). At a P-tau217 cutoff level of < 0.829 pg/mL, specificity of ≥ 90% was achieved for excluding of A+T_PET_high_, which resulted however in a poor sensitivity of 33% for identifying A+T_PET_low/int_ (Fig. 2D). Participants with P-tau217 values < 0.612 pg/mL were classified as A-T_plasma_P−tau217_low_; participants with P-tau217 values from 0.612 pg/mL to 0.829 pg/mL were classified as A+T_plasma_P−tau217_low/int_; participants with P-tau217 values > 0.829 pg/mL were classified as A+T_plasma_P−tau217_high_. The AT_plasma_P−tau217_stages_, compared to the original AT_PET_stages_ is visualized as a Sankey and Prevalence bar plot in supplementary Fig. 1A and B. Overall, 34 participants were classified as A-T_plasma_P−tau217_low_ (25 true A-T_PET_low_), 10 as A+T_plasma_P−tau217_low/int_ (6 true A+T_PET_low/int_) and 55 as the A+T_plasma_P−tau217_high_ (23 true A+T_PET_high_).

Although group-level ROC statistics did not imply benefit for combining plasma marker results, we did continue to explore individual benefit of the backward-selected two-marker combinations. Applying ≥ 90% specificity cutoffs on the predicted logistic regression probability values of our two-marker combinations (P-tau217 and Aβ42/40 for A-T_PET_low_ versus A+T_PET_low/int_, and P-tau217 and P-tau181 for A+T_PET_low/int_ versus A+T_PET_high_), resulted in the following re-classifications: 33 participants were classified as A-T_plasma_2marker_low_ (25 true A-T_PET_low_), 22 as A+T_plasma_2marker_low/int_ (18 true A+T_PET_low/int_) and 43 as A+T_plasma_2marker_high_ (23 true A+T_PET_high_) (supplementary Fig. 1). Compared to P-tau217 alone, the two-marker combinations thus resulted in a higher number of correctly classified A+T_low/int_ participants, and a lower number of falsely classified A+T_high_ participants.

### Association of AT_plasma_stages_ groups versus AT_PET_stages_ groups with cognitive trajectories

**Fig. 3:**
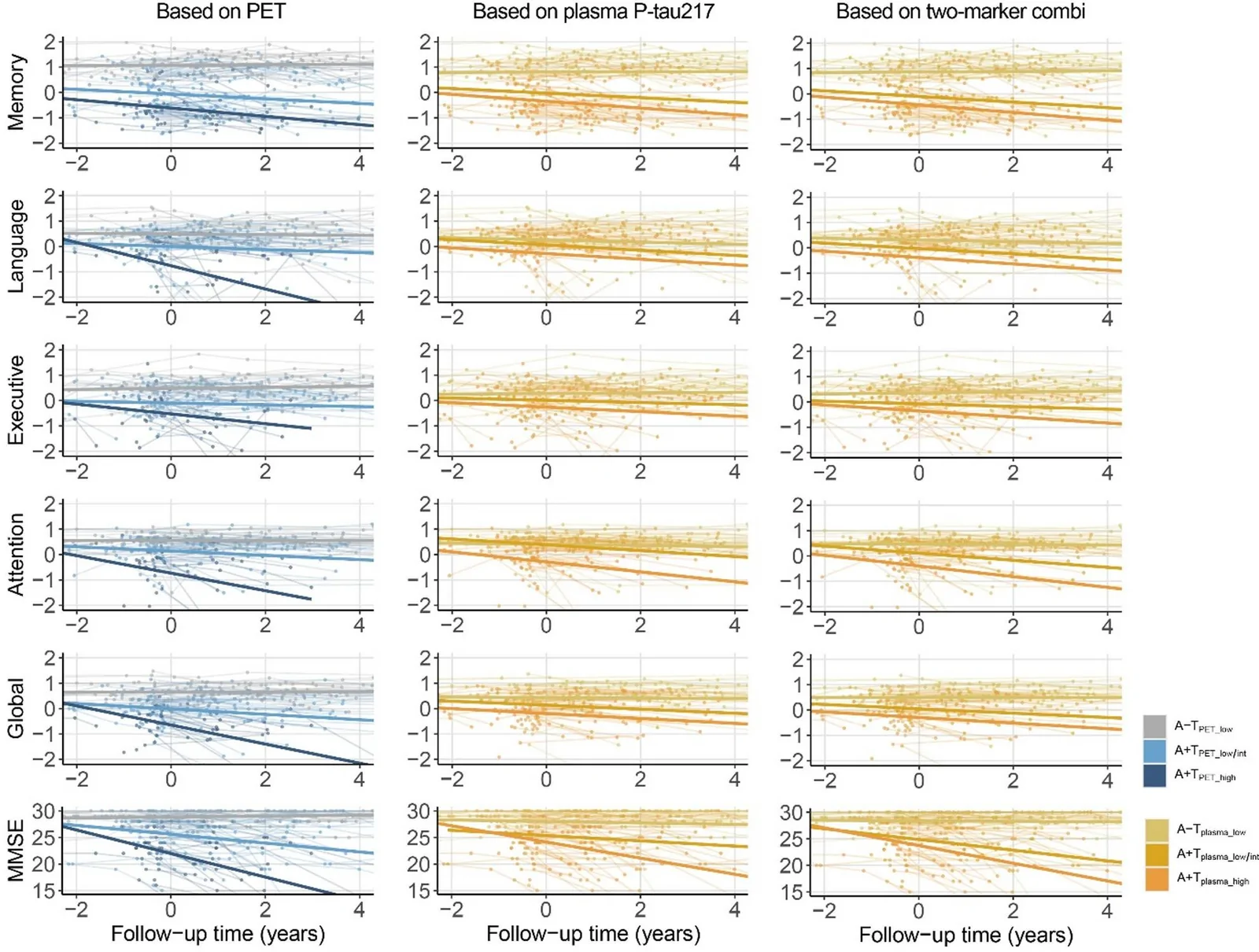
Spaghetti plots visualizing the association of AT_PET_stages_ (left), AT_plasma_P-tau217_stages_ (middle) and AT_plasma_2-marker_stages_ (right) with longitudinal cognitive performance across domains, global cognition and MMSE. A = amyloid, T = tau, Int = intermediate, MMSE = mini-mental state examination

**Fig. 4:**
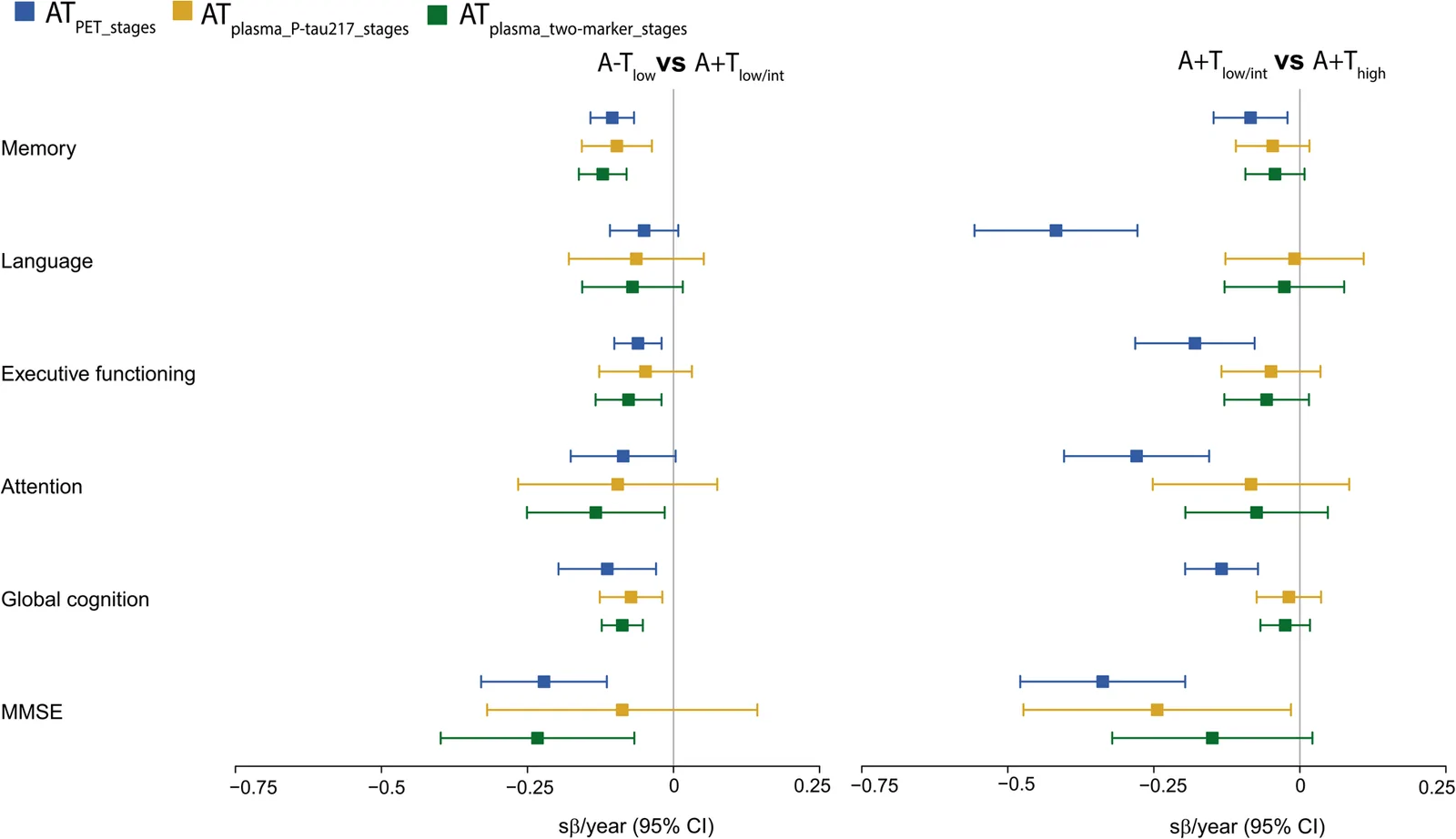
Coefficient plots visualizing the associations of AT_PET_stages_ groups (blue), AT_plasma_P−tau217_stages_ groups (yellow) and AT_plasma_2−marker_stages_ groups (green) with longitudinal cognitive performance across cognitive domains, global cognition and MMSE. The standardized beta (sβ) represents the yearly standard deviation change in neuropsychological test performance for subsequent groups. Int = intermediate, MMSE = Mini-Mental State Examination

To examine the possible clinical meaningfulness of the developed AT_plasma_P−tau217_stages_ and AT_plasma_2marker_stages_ in comparison to the original AT_PET_stages_, we compared the associations of the two plasma groupings and the PET grouping with concurrent and longitudinal cognitive performance across MMSE, global cognition and relevant cognitive domains (supplementary Table 1, supplementary Table 2, Figs. 3 and 4; exploratory associations of AT_plasma_5marker_stages_ with cognitive trajectories: supplementary Fig. 2 and supplementary Table 2; exploratory associations of AT_PET_stages_ with A+T_PET_low_ and A+T_PET_int_ groups separate: supplementary Fig. 3).

For the AT_PET_stages_, we observed clear differentiation between the subsequent groups A-T_PET_low_ versus A+T_PET_low/int_ and A+T_PET_low/int_ versus A+T_PET_high_ groups in both their concurrent and longitudinal cognitive performance scores. Specifically, linear mixed models showed that A+T_PET_low/int_ participants had worse concurrent MMSE, global cognition and cognitive domain scores across all domains than A-T_PET_low_ participants (range sβ -0.44 – -1.14, all *p* < 0.05^FDR^), and likewise, A+T_PET_high_ participants had worse concurrent MMSE, global cognition and cognitive domain scores across all domains than A+T_PET_low/int_ participants (range sβ -0.55 – -0.94, all *p* < 0.01^FDR^). A+T_PET_low/int_ participants had steeper rates of decline on scores for MMSE, global cognition, memory and executive functioning (range sβ/year -0.06 – -0.22, all *p* < 0.05^FDR^) than A-T_PET_low_ participants, and A+T_PET_high_ participants had steeper rates of decline on scores for MMSE, global cognition and cognitive domain scores across all domains than A+T_PET_low/int_ participants (range sβ/year -0.08 – -0.42, all *p* < 0.05^FDR^).

By contrast, for the AT_plasma_P−tau217_stages_, we observed no clear differentiation between the subsequent groups A-T_plasma_P-tau217_low_ versus A+T_plasma_P-tau217_low/int_ and A+T_plasma_P-tau217_low/int_ versus A+T_plasma_P-tau217_high_, neither in their concurrent nor in their longitudinal cognitive performance scores. Specifically, the linear mixed models showed that only concurrent memory performance was worse for A+T_plasma_P-tau217_low/int_ participants compared to A-T_plasma_P-tau217_low_ participants (sβ=-0.89, SE = 0.27, *p* < 0.01^FDR^), and there were no differences in concurrent cognitive performance between A+T_plasma_P-tau217_low/int_ and A+ T_plasma_P-tau217_high_ groups. Furthermore, only differences in rates of decline in global cognition and memory scores were observed between the A-T_plasma_P-tau217_low_ and A+T_plasma_P-tau217_low/int_ participants (sβ/year=-0.07 and sβ/year=-0.10, respectively; both: *p* < 0.01^FDR^), and there was no difference in rates of decline in global cognition nor in cognitive domain scores across all domains between the A+T_plasma_P-tau217_low/int_ and the A+T_plasma_P-tau217_high_ participants.

Compared to AT_plasma_stages_ based on only plasma P-tau217, the two-marker combination staging approach showed clearer differentiation between participants classified as A-T_plasma_2marker_low_ and participants classified as A+T_plasma_2marker_low/int_. Linear mixed models showed that concurrent MMSE, global cognition, memory and executive functioning were significantly worse in A+T_plasma_2marker_low/int_ compared to A-T_plasma_2marker_low_. Also rates of decline in MMSE, global cognition, memory, executive functioning and attention were significantly steeper in A+T_plasma_2marker_low/int_ compared to A-T_plasma_2marker_low_. Similarly to the AT_plasma_P-tau217_stages_, there was no differentiation in concurrent nor longitudinal cognitive performance scores between A+T_plasma_2marker_low/int_ and A+T_plasma_2marker_high_. Exploratively examining relationships between AT_plasma_5marker_stages_ and concurrent and longitudinal cognitive performance scores did not result in any improved separation of clinical trajectories between the AT groups compared to the AT_plasma_2marker_stages_ nor compared to the AT_plasma−P-tau217_stages_ groupings.

## Discussion

The present study examined the performance of the AD-related plasma biomarkers P-tau217, P-tau181, Aβ42/40, GFAP and NfL measured on an immunoassay platform with scalability for ultimate clinical implementation to biologically stage amyloid status (+/-) together with tau-PET burden (low, intermediate, high). Our ROC-AUC results suggest that plasma P-tau217 is effective to discriminate amyloid negative individuals (sensitivity = 80% at specificity ≥ 90%; AUC = 0.92), while the ability of plasma P-tau217 to discriminate amyloid positive individuals with a high tau-PET burden stage is only moderate (sensitivity = 33% at specificity ≥ 90%; AUC = 0.74). Our subsequent analyses associating plasma P-tau217-based severity stages with longitudinal cognition confirmed that a plasma-based staging approach with P-tau217 did not equal tau PET-based severity staging. Where more advanced tau PET-based severity stages associated with worse cognitive performance and steeper rates of cognitive decline, plasma P-tau217-based severity stages were not so clearly associated with cognitive performance and decline. Further, on the group level, diagnostic differentiation (AUC) did not statistically improve when two markers selected with a backward logistic regression approach were combined (*i.e.*, P-tau217 with Aβ42/40 and P-tau217 with P-tau181). However, when applying such marker combination for reclassification and subsequent prediction of rates of cognitive decline, we observed that more participants were correctly classified as intermediate tau burden, and in agreement, cognitive trajectories were more differentiated with this 2-marker based A-T_low_ and A+T_low/int_ staging approach as compared to staging with only P-tau217. Our results suggested that non-AD specific biomarkers NfL and GFAP did not improve concordance with tau-PET stages among amyloid positive individuals. In addition, utilizing all 5 markers to predict cognitive slopes was not more precise than utilizing P-tau217 or our identified 2-marker combinations including P-tau217, P-tau181 and Aβ42/40. Still, our plasma-based staging approaches did not equal tau-PET, especially in differentiating clinical trajectories of A+T_low/int_ and A+T_high_. This suggests that additional, novel plasma biomarkers need to be identified and incorporated to improve AD biological staging through blood testing. A plasma biomarker-based biological staging approach as replacement of tau-PET would be of great clinical benefit, due to its accessibility and scalability, and has the potential to significantly streamline patient inclusions in clinical trials and clinical decision making on initiating amyloid-targeting therapies.

Most previous studies have primarily looked into the utility of blood-based biomarkers P-tau217, P-tau181, Aβ42/40, GFAP and NfL for binary classification of amyloid status [46–48] or tau status [49–52]. We combined amyloid status and tau staging in our study, since a refined diagnostic approach to AD severity staging will be crucial to optimize treatment strategies, according to recent trial results where low or intermediate tau burden was associated with more favorable clinical outcomes [5, 53, 54]. In line with previous work [48], we found that from all core AD-related plasma markers, plasma P-tau217 is the strongest predictor of amyloid status (in our study: AUC = 0.92). Also in line with previous work, plasma P-tau217 had the highest discriminatory performance for identifying high tau-PET burden among amyloid positive individuals (in our study: AUC = 0.74) [13]. The overlap in P-tau217 biomarker levels across intermediate and high tau-PET stages was however considerable, resulting in a very low sensitivity. This was earlier reported by others as well [13–15, 55]. Illustratively, a head-to-head study comparing tau-PET SUVR and plasma P-tau217 concentrations among amyloid positive individuals with MCI and mild dementia found an overall good correlation coefficient of *r* = 0.61 between the two measures, but simultaneously revealed substantial heterogeneity in regional patterns across individuals [16]. This is evident from our Sankey and prevalence plots (supplementary Fig. 1), which show that, when applying the developed cutoffs in our cohort, only 6 out of 47 A+T_PET_low/int_ participants with valid biomarker results were correctly classified as A+T_PET_low/int_ based on their plasma results. Of note, in our cohort, we could marginally improve this classification by including Aβ42/40 and P-tau181 results.

In agreement with our limited concordance between the PET-based and the developed plasma-based biological severity stages, among amyloid-positive individuals, clinical trajectories differed by tau-PET staging group (low/intermediate vs. high tau burden), but not by plasma staging groups (independent of the staging method applied; P-tau217 alone, 2-marker combination or 5-marker combination). Those who were low/intermediate in tau-PET burden and those who were high in tau-PET burden showed clearly distinct clinical trajectories. Previous studies showed that plasma P-tau217 and tau-PET can predict cognitive decline in cognitively unimpaired individuals, on the group level [56], but tau-PET shows superiority in predicting cognitive decline among cognitively impaired individuals [57, 58]. Of note, severity stage subgroups based on CSF P-tau and tau levels were previously also found to be associated with cognitive state and cognitive decline [9, 59]. Our approach, applying our developed staging approaches to longitudinal clinical data, highlighted the importance of looking further than AUCs when assessing clinical utility. Based on AUC alone, performance of our plasma staging schemes seemed moderate (AUC > 0.74), but looking further into classification accuracy and the prediction of clinical trajectories based on our developed staging scheme, highlighted the differences in diagnostic sensitivity of our plasma-based versus the PET-based staging schemes. By incorporating clinical trajectories in our study design, we better captured clinical heterogeneity between individuals, which is essential when moving from group level statistics to assessment of individual utility. 

Interestingly, Wald’s backwards elimination logistic regression selected P-tau181 and Aβ42/40 next to P-tau217 to improve discrimination of amyloid negative participants or amyloid positive participants with a high tau PET burden. However, the resulting AUCs of the marker combinations were only slightly higher compared to plasma P-tau217 alone. For simplicity of interpretation, logistical feasibility and clinical adoption, simpler models with less plasma biomarkers may be preferred. Hence we decided to continue our analyses also with only plasma P-tau217, and presented those findings next to our two-marker combinations. All our results viewed in aggregate, suggest that we might want to look further than the most validated plasma markers to date (P-tau217, P-tau181, Aβ42/40, GFAP and NfL), and should extend the plasma staging model with markers in addition to P-tau217, to better capture the spatiotemporal pattern of AD as we capture it on tau-PET scans. Future research would benefit from investigating other P-tau isoforms. A recently identified plasma marker that tracks with tau-PET burden and cognitive trajectories is plasma MTBR-tau243 [60]. Thus far, this plasma biomarker can only be measured using targeted mass spectrometry, not yet with clinical immunoassays. The development of clinical immunoassays will be essential for commutability and scalability of the microtubule binding region (MTBR)-tau243 plasma test. Development of a robust assay for MTBR-tau243 is not trivial, due to the highly conserved and structurally compact nature of this region of the tau protein, which hinders antibody accessibility (target epitope ~ 11 amino acids). Additionally, the low MTBR-tau243 plasma concentration challenges the sensitivity limits of immunoassay-based detection methods. Another potential marker may be P-tau205 or non-phosphorylated brain-derived tau forms, which were also suggested to better track with tau-PET burden and cognitive trajectories [61] than P-tau217 and the other markers investigated here, which all seem to associate more closely with amyloid burden than with tau burden.

Our study employed the MUBADA ROI for tau-PET staging, which was also used for patient inclusion in the Donanemab clinical trial, where they showed that participants with low/intermediate tau burden gained greater clinical benefit from treatment than participants with high tau burden [3, 5]. While the Donanemab clinical trial implemented tau-PET staging in its inclusion criteria, recommendations by the FDA and EMA, as well as according to appropriate use recommendations presented by experts in the field [62], do not mention that tau-PET (staging) is required to initiate Donanemab and Lecanemab treatment [62–64]. Access and affordability of tau-PET form its evident challenges, but accurately identifying participants best suited for treatment remains crucial, considering the potential side effects of anti-amyloid therapies like ARIA (amyloid-related imaging abnormalities), and may be recommendable as good clinical practice (whether tau-PET-based, or plasma-based or CSF-based). In addition, while clinical trial results demonstrate beneficial effects in participants with low/medium tau levels [5], caution is advised when including amyloid positive participants with low tau. Although these individuals may be in the early stages of AD, this biomarker profile could also indicate the presence of comorbidities contributing to memory impairment [65], especially in individuals with a worse clinical stage compared to their biological stage (*i.e.*, low tau burden but advanced cognitive impairment) [39, 66]. To fully capture an individual’s biological AD stage, it is also essential to consider a broader range of pathological processes beyond amyloid and tau. Markers of synaptic dysfunction, vascular integrity and co-pathologies (e.g. TDP-43, a-synuclein) provide complementary insights into the underlying heterogeneity of the disease. Proteomics-based approaches may be helpful to identify staging biomarkers reflecting those pathophysiological processes.

Limitations of this study include our relatively small sample size, which was drawn from a single memory clinic cohort. This cohort had a relatively high proportion of participants with a high tau PET burden, reflecting our clinic’s specialty in early-onset and atypical AD, but thereby limiting the generalizability of our findings. Moreover, our cohort included cognitively unimpaired participants with and without AD pathology, which does not reflect the current population intended to receive anti-amyloid treatment. However, including cognitively unimpaired amyloid positive participants remains valuable, as the clinical trial landscape is now shifting towards early intervention in the preclinical stage of AD [67]. Another study limitation is the lack of measurement of more aggregated tau-specific biomarkers like MTBR-tau243 and p-tau205 [60]. Lastly, the plasma samples were collected at only one time point per participant, while longitudinal plasma results may enhance its tau-PET staging performance.

## Conclusions

In conclusion, our study showed that plasma P-tau217 offers good diagnostic sensitivity for discriminating amyloid negative participants who are not suited to receive anti-amyloid treatments. Tau staging in amyloid-positive individuals remains optimally performed with tau-PET scans, until a sensitive staging scheme can be developed incorporating accessible biomarkers that more accurately reflect neurofibrillary tangle burden.

## Supplementary Information

Supplementary Material 1.

## Data Availability

The dataset generated during the current study is stored as part of the Amsterdam Dementia Cohort, and is available from the corresponding author on reasonable request for collaborative research efforts.

## Abbreviations

AD: Alzheimer’s disease
Aβ: Amyloid-β
P-tau: Hyperphosphorylated tau
PET: Positron emission tomography
CSF: Cerebrospinal fluid
GFAP: Glial fibrillary acidic protein
NfL: Neurofilament light
MUBADA: Multiblock barycentric discriminant analysis
SCD: Subjective cognitive decline
MCI: Mild cognitive impairment
VOI: Volume of interest
ROC: Receiver operating characteristics
AUC: Area under the curve
SUVR: Standardized uptake value ratio
A: Amyloid status
T: Tau status
FDR: False discovery rate
MTBR: Nicrotubule binding region
