# Supplementary material for: The potential of key Alzheimer’s plasma biomarkers to mimic tau PET MUBADA-based disease staging
Source: Alzheimers Res Ther. 2026 Jun 30;18:206. doi: 10.1186/s13195-026-02085-6 (PMC13591876; doi:10.1186/s13195-026-02085-6)
Supplement: Supplementary file 1 [file 13195_2026_2085_MOESM1_ESM.pdf]

SUPPLEMENTARY FILE

The potential of Alzheimer's plasma biomarkers to mimic tau PET MUBADA-based disease staging

Inge M.W. Verberk, PhD[1,4*†]; Lotte A. de Koning, MSc[2,6*]; Emma M. Coomans, PhD[3,4]; Calvin Trieu, MD[1,3,4]; Anna E. Leeuwis, PhD[3,4,5]; Jacob Hunter[7]; Lee Honigberg[7]; Wiesje M. van der Flier, PhD[4,8,9,10]; Everard G.B. Vijverberg, MD, PhD[3,4]; Rik Ossenkoppele, PhD[3,4]; Elsmarieke van de Giessen, MD, PhD[2,6]; Charlotte E. Teunissen, PhD[1,4]

** Shared first authorship*

[1] Neurochemistry Laboratory, Department of Laboratory Medicine, Amsterdam UMC, Vrije Universiteit Amsterdam, Amsterdam, The Netherlands

[2] Department of Radiology and Nuclear medicine, Amsterdam UMC, Vrije Universiteit Amsterdam, Amsterdam, The Netherlands

[3] Alzheimer Center, Department of Neurology, Amsterdam UMC, Vrije Universiteit Amsterdam, Amsterdam, The Netherlands

[4] Amsterdam Neuroscience, Neurodegeneration, Amsterdam, The Netherlands

[5] Department of Medical Psychology, Amsterdam UMC, Vrije Universiteit Amsterdam, Amsterdam, the Netherlands.

[6] Amsterdam Neuroscience, Brain Imaging, Amsterdam, The Netherlands

[7] ALZpath Inc, California, USA

[8] Alzheimer Nederland, Amersfoort, the Netherlands

[9] Epidemiology and Data Science, Vrije Universiteit Amsterdam, Amsterdam UMC location VUmc, Amsterdam, The Netherlands

[10] Amsterdam Public Health, Amsterdam, the Netherlands

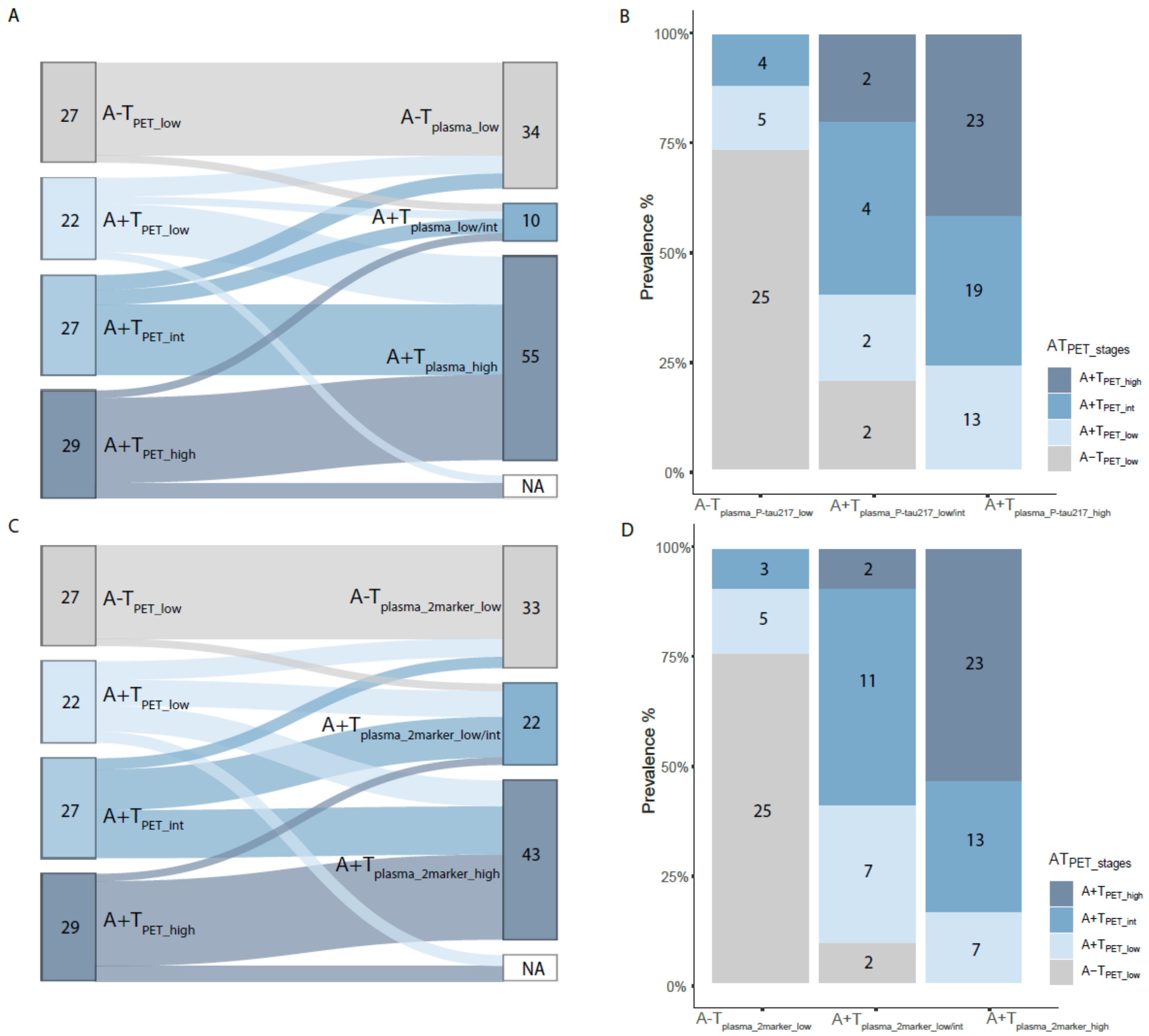

Supplementary figure 1. Sankey plot (A/C) and prevalence plot (B/D) visualizing the AT_{PET_stages} groups versus the developed $AT_{plasma\_P\text{-}tau217\_stages}$ (top) and $AT_{plasma\_2marker\_stages}$ (bottom) groups. *The AT_{PET_stages} groups are based on amyloid (A) status (-/+) according to cerebrospinal fluid testing or amyloid PET and tau (T) according to positron emission tomography (PET) scanning, where the tau-PET scan was interpreted according to the MUBADA SUVr and visual read. The $AT_{plasma\_P\text{-}tau217\_stages}$ groups are based on the plasma P-tau217 levels predicting the AT_{PET_stages}, with thresholds set at >90% specificity. The $AT_{plasma\_P\text{-}tau217\_stages}$ groups are based on the two-marker combinations (P-tau217 and Aβ42/40 for A-T_{PET_low} versus A+$T_{PET\_low/int}$, and P-tau217 and P-tau181 for A+$T_{PET\_low/int}$ versus A+T_{PET_high}) predicting the AT_{PET_stages}) with thresholds set at >90% specificity. 6 participants are classified as not available (NA) in* $AT_{plasma\_P\text{-}tau217\_stages}$ groups and 7 participants are classified as NA in $AT_{plasma\_2marker\_stages}$ *due to missing plasma biomarker levels. Int = intermediate.*

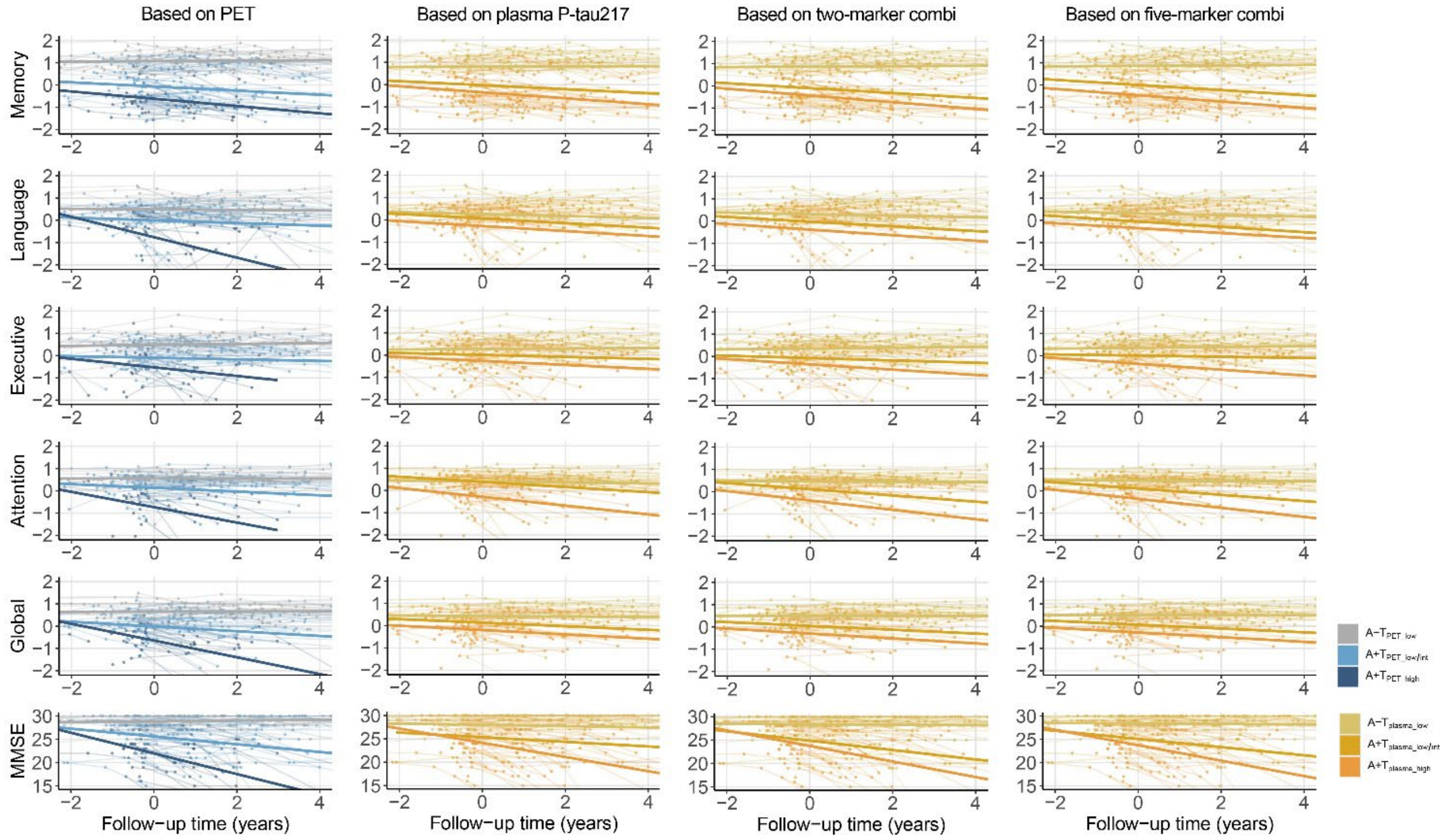

Supplementary figure 2. Spaghetti plots visualizing the association of AT$_{PET_stages}$ (first column), AT$_{plasma_P\text{-}tau217_stages}$ (second column), AT$_{plasma_2\text{-}marker_stages}$ (third column) and AT$_{plasma_5marker_stages}$ (right column) with longitudinal cognitive performance across domains, and global cognition and MMSE.

A = amyloid, T = tau, Int = intermediate, MMSE = mini-mental state examination.

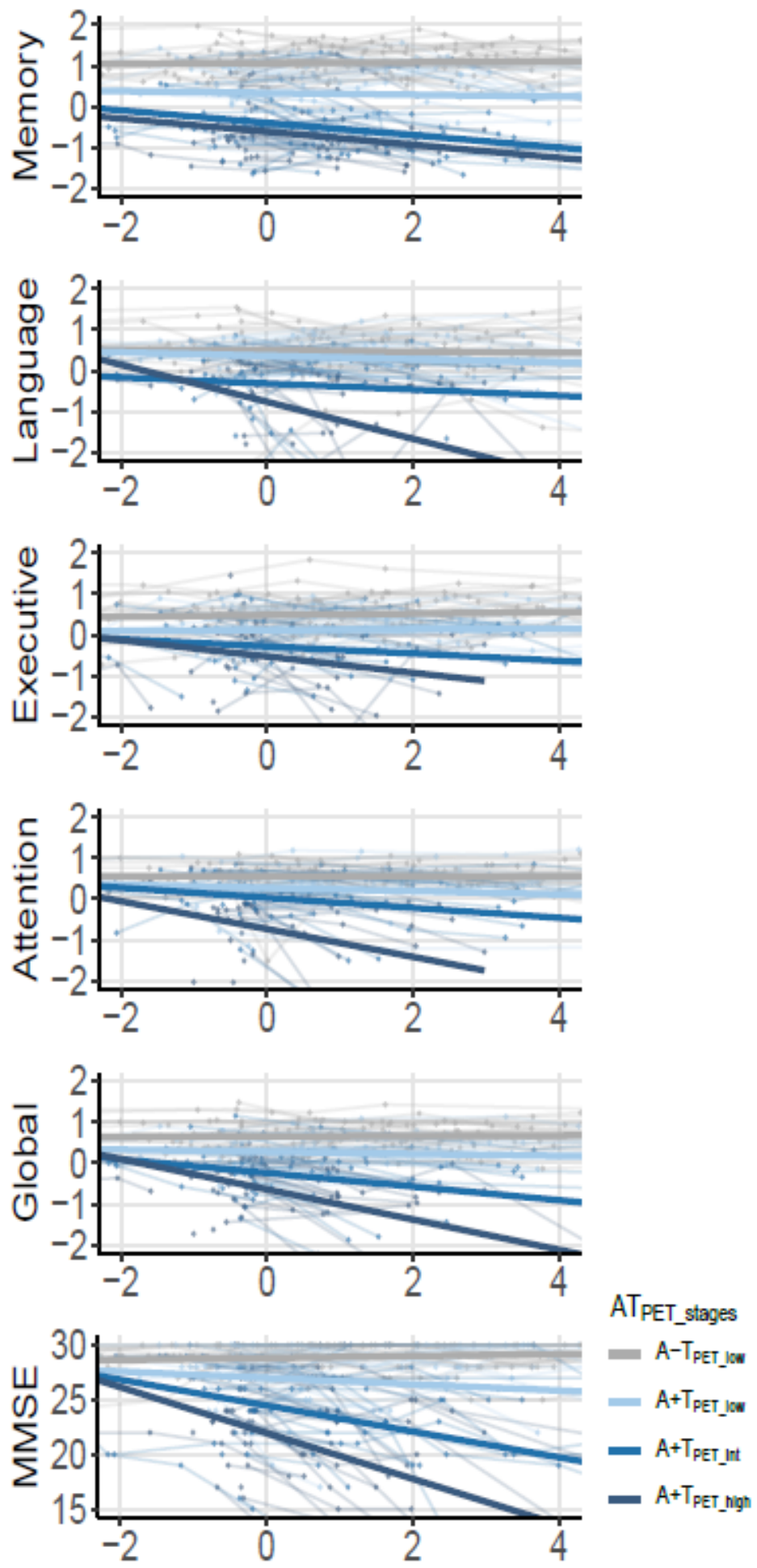

Supplementary figure 3. Spaghetti plots visualizing the association of AT_{PET_stages} with longitudinal cognitive performance across domains, and global cognition and MMSE, where AT_{PET_stages} are splitted across four groups: A-T_{PET_low}, A+T_{PET_low}, A+T_{PET_int} and A+T_{PET_high}.

A = amyloid, T = tau, Int = intermediate, MMSE = mini-mental state examination.

Supplementary table 1. Associations of $AT_{plasma\_P\text{-}tau217\_stages}$ and AT_{PET_stages} with cross-sectional and longitudinal cognitive performance across cognitive domains and global cognition.

	$A\text{-}T_{PET\_low}$ vs $A\text{+}T_{PET\_low/int}$		$A\text{+}T_{PET\_low/int}$ vs $A\text{+}T_{PET\_high}$		$A\text{-}T_{plasma\_P\text{-}tau217\_low}$ vs $A\text{+}T_{plasma\_P\text{-}tau217\ low/int}$		$A\text{+}T_{plasma\_P\text{-}tau217\_low/int}$ vs $A\text{+}T_{plasma\_P\text{-}tau217\_high}$	
Cognitive domain	Concurrent	Annual change	Concurrent	Annual change	Concurrent	Annual change	Concurrent	Annual change
Memory	**-1.14[0.16]*** **	**-0.10[0.02]*** **	**-0.60[0.17]*** **	**-0.08[0.03]** **	**-0.89[0.27]** **	**-0.10[0.03]* **	-0.27[0.26]	-0.05[0.03]
Language	**-0.49[0.21]* **	-0.05[0.03]	**-0.81[0.22]*** **	**-0.42[0.07]*** **	-0.24[0.35]	-0.06[0.06]	-0.30[0.33]	-0.01[0.06]
Executive functioning	**-0.58[0.15]*** **	**-0.06[0.02]** **	**-0.55[0.17]** **	**-0.18[0.05]*** **	-0.36[0.26]	-0.05[0.04]	-0.21[0.25]	-0.05[0.04]
Attention	**-0.44[0.18]* **	-0.09[0.04]	**-0.88[0.19]*** **	**-0.28[0.06]*** **	-0.05[0.31]	-0.10[0.08]	-0.65[0.30]*	-0.08[0.08]
Global cognition	**-0.67[0.13]*** **	**-0.11[0.04]* **	**-0.65[0.13]*** **	**-0.13[0.03]*** **	-0.39[0.22]	**-0.07[0.03]* **	-0.27[0.21]	-0.02[0.03]
MMSE	**-0.78[0.19]*** **	**-0.22[0.05]*** **	**-0.94[0.20]*** **	**-0.34[0.07]*** **	-0.71[0.32]*	-0.09[0.11]	-0.24[0.30]	-0.24[0.11]*

*Standardized β and standard error of linear mixed models associating AT_{PET_stages} (left) or $AT_{Plasma\_P\text{-}tau217\_stages}$ (right) groups with longitudinal cognitive domain scores, corrected for age, sex and education, are presented. The standardized beta represents either the cross-sectional difference in cognitive performance at time of the PET imaging, or the annual standard deviation change in cognitive performance, for subsequent stages. A = amyloid status, Int = intermediate, In bold font, *p<0.05^{FDR}, **p<0.01 ^{FDR}, ***p<0.001 ^{FDR}; in regular font, *p<0.05 prior to FDR correction.*

Supplementary table 2. Associations of AT_{plasma_stages} based on 2-marker and 5-marker combinations with cross-sectional and longitudinal cognitive performance across cognitive domains and global cognition.

	$A\text{-}T_{plasma\_low}$ vs $A\text{+}T_{plasma\_int}$ *Based on 2-marker combination*		$A\text{+}T_{plasma\_int}$ vs $A\text{+}T_{plasma\_high}$ *Based on 2-marker combination*		$A\text{-}T_{plasma\_low}$ vs $A\text{+}T_{plasma\_int}$ *Based on 5-marker combination*		$A\text{+}T_{plasma\_int}$ vs $A\text{+}T_{plasma\_high}$ *Based on 5-marker combination*	
Cognitive domain	Concurrent	Annual change	Concurrent	Annual change	Concurrent	Annual change	Concurrent	Annual change
Memory	**-1.01[0.21]*** **	**-0.12[0.02]*** **	-0.28[0.21]	-0.04[0.03]	**-0.87[0.21]*** **	**-0.12[0.02]*** **	-0.46[0.20]*	-0.03[0.06]
Language	-0.39[0.27]	-0.07[0.04]	-0.29[0.27]	-0.03[0.05]	-0.39[0.27]	-0.08[0.04]	-0.29[0.27]	-0.001[0.05]
Executive functioning	**-0.45[0.20]* **	**-0.08[0.03]* **	-0.28[0.20]	-0.06[0.04]	-0.35[0.09]	-0.05[0.03]	-0.41[0.20]*	**-0.10[0.04]** **
Attention	-0.39[0.23]	**-0.13[0.05]* **	-0.46[0.23]*	-0.07[0.06]	-0.38[0.24]	-0.13[0.06]*	-0.45[0.23]	-0.07[0.06]
Global cognition	**-0.50[0.17]** **	**-0.09[0.02]*** **	-0.29[0.17]	-0.03[0.02]	**-0.46[0.17]* **	**-0.09[0.02]*** **	-0.34[0.17]*	-0.03[0.02]
MMSE	**-0.91[0.24]*** **	**-0.23[0.08]* **	-0.23[0.23]	-0.15[0.08]	**-0.83[0.24]*** **	**-0.20[0.09]* **	-0.32[0.24]	-0.18[0.09]*

*Standardized β and standard error of linear mixed models associating AT_{plasma_stages} based on two-marker combinations (left) or all-5-marker combinations (right) groups with longitudinal cognitive domain scores, corrected for age, sex and education, are presented. The standardized beta represents either the cross-sectional difference in cognitive performance at time of the PET imaging, or the annual standard deviation change in cognitive performance, for subsequent stages. The two-marker combinations employed P-tau217 + Aβ42/40 to discriminate $A\text{-}T_{PET\_low}$ from $A\text{+}T_{PET\_low}$ and P-tau217 + P-tau181 to discriminate $A\text{+}T_{PET\_high}$ from $A\text{+}T_{PET\_low/int.}$ The 5-marker combination included P-tau217, P-tau181, Aβ42/40, GFAP and NfL. Predicted values from logistic regression models combining the plasma biomarkers were used in the linear mixed models. A = amyloid status, Int = intermediate, In bold font, *p<0.05^{FDR}, **p<0.01 ^{FDR}, ***p<0.001 ^{FDR}; in regular font, *p<0.05 prior to FDR correction.*
